# Selective κ receptor partial agonist HS666 produces potent antinociception without inducing aversion after i.c.v. administration in mice

**DOI:** 10.1111/bph.13854

**Published:** 2017-06-27

**Authors:** Mariana Spetea, Shainnel O Eans, Michelle L Ganno, Aquilino Lantero, Michael Mairegger, Lawrence Toll, Helmut Schmidhammer, Jay P McLaughlin

**Affiliations:** ^1^ Department of Pharmaceutical Chemistry, Institute of Pharmacy and Center for Molecular Biosciences Innsbruck (CMBI) University of Innsbruck Innsbruck Austria; ^2^ Torrey Pines Institute for Molecular Studies Port St. Lucie FL USA; ^3^ Department of Pharmacodynamics University of Florida Gainesville FL USA

## Abstract

**Background and purpose:**

The κ receptor has a central role in modulating neurotransmission in central and peripheral neuronal circuits that subserve pain and other behavioural responses. Although κ receptor agonists do not produce euphoria or lead to respiratory suppression, they induce dysphoria and sedation. We hypothesized that brain‐penetrant κ receptor ligands possessing biased agonism towards G protein signalling over β‐arrestin2 recruitment would produce robust antinociception with fewer associated liabilities.

**Experimental approach:**

Two new diphenethylamines with high κ receptor selectivity, HS665 and HS666, were assessed following i.c.v. administration in mouse assays of antinociception with the 55°C warm‐water tail withdrawal test, locomotor activity in the rotorod and conditioned place preference. The [^35^S]‐GTPγS binding and β‐arrestin2 recruitment *in vitro* assays were used to characterize biased agonism.

**Key results:**

HS665 (κ receptor agonist) and HS666 (κ receptor partial agonist) demonstrated dose‐dependent antinociception after i.c.v. administration mediated by the κ receptor. These highly selective κ receptor ligands displayed varying biased signalling towards G protein coupling *in vitro*, consistent with a reduced liability profile, reflected by reduced sedation and absence of conditioned place aversion for HS666.

**Conclusions and implications:**

HS665 and HS666 activate central κ receptors to produce potent antinociception, with HS666 displaying pharmacological characteristics of a κ receptor analgesic with reduced liability for aversive effects correlating with its low efficacy in the β‐arrestin2 signalling pathway. Our data provide further understanding of the contribution of central κ receptors in pain suppression, and the prospect of dissociating the antinociceptive effects of HS665 and HS666 from κ receptor‐mediated adverse effects.

**Abbreviations**
CPA: conditioned place aversion
CPP: conditioned place preference
DAMGO: [d‐Ala^2^,*n*‐MePhe^4^, Gly‐ol]enkephalin
N/OFQ: nociceptin/orphanin FQ
NOP: nociceptin opioid
nor‐BNI: nor‐binaltorphimine
U50,488: (±)‐*trans*‐3,4‐dichloro‐*N*‐methyl‐*N*‐[2‐(1‐pyrrolidinyl)cyclohexyl]benzeneacetamide
U69,593: (+)‐(5α,7α,8β)‐(−)*N*‐methyl‐*N‐*[7‐(1‐pyrrolidinyl)‐1‐oxaspiro(4,5)dec‐8‐yl]benzeneacetamide

## Introduction

The κ receptor, a GPCR activated by the dynorphins, shares extensive homology with the other members of the opioid receptor family, μ , δ and nociceptin opioid (NOP) receptors, but has distinct pharmacological and physiological effects (Lemos and Chavkin, 2011; Cahill *et al.*, 2014; Lalanne *et al.*, 2014). Given the significance of the dynorphin/κ receptor system as a powerful regulator of many physiological and behavioural responses (i.e. pain, stress, motivation, emotion, cognition and reward), the κ receptor is a key target for developing pharmacotherapies for neuropsychiatric disorders (Aldrich and McLaughlin, 2009; Kivell and Prisinzano, 2010; Butelman *et al.*, 2012; Carroll and Carlezon, 2013; Spetea *et al.*, 2013a; Urbano *et al.*, 2014).

The μ receptor agonists are efficacious analgesics (Spetea *et al.*, 2013b; Pasternak, 2014) but induce unwanted euphoria, addiction, respiratory depression and gastrointestinal tract inhibition (Benyamin *et al.*, 2008; Atkinson *et al.*, 2014). The κ receptor modulates pain processing at central and peripheral sites, and pain remains a likely indication for κ agonists (Kivell and Prisinzano, 2010; Albert‐Vartanian *et al.*, 2016). Moreover, κ agonists do not cause the liabilities of μ receptor activation. Animal and human studies with brain‐penetrating κ agonists (i.e. U50,488, salvinorin A, spiradoline, enadoline and asimadoline) established other dose‐limiting neuropsychiatric side effects including sedation, dysphoria and psychotomimesis attributed to κ receptors in the CNS, limiting their further development as analgesics (Kivell and Prisinzano, 2010; Albert‐Vartanian *et al.*, 2016). Recent evidence suggests that the κ receptor‐mediated antinociception results from G protein‐mediated signalling events, while signalling through alternative pathways (i.e. β‐arrestin2) promotes negative side effects (Bruchas and Chavkin, 2010; Dogra and Yadav, 2015; Rankovic *et al.*, 2016). Thus, the concept of biased agonism at the κ receptor has gained significance in drug discovery, where the development of G protein‐biased κ receptor agonists may deliver the desired analgesia while avoiding deleterious mood disturbances.

**Figure 1:**
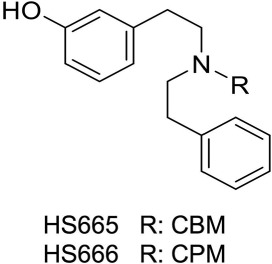
Structures of HS665 and HS666. CBM, cyclobutylmethyl; CPM: cyclopropylmethyl.

Our recent research led to a series of structurally distinct κ receptor ligands from the class of diphenethylamines (Spetea *et al.*, 2012; Guerrieri *et al.*, 2015; Guerrieri *et al.*, 2016). We previously carried out structure–activity relationship studies based on *in vitro* biological properties and molecular modelling and reported that distinct variations, such as the nature of the substituent at the nitrogen or the position of the phenolic hydroxyl group, can modulate binding and selectivity for the κ receptor and agonist–antagonist activities (Spetea *et al.*, 2012; Guerrieri *et al.*, 2016), but a detailed investigation of the pharmacology of these compounds was warranted. Presently, HS665 (Figure 1) was selected because its human κ receptor affinity (0.49 nM) was found to be the highest in the series, and it was the only full κ receptor agonist. Our initial *in vivo* work established κ receptor‐mediated antinociceptive effects of HS665 comparable with U50,488 after s.c. administration to mice in the writhing test. The *N*‐cyclopropylmethyl‐substituted analogue HS666 (Figure 1) was also evaluated, based on its high affinity for the κ receptor (5.90 nM) and potent partial agonism of the κ receptor (Spetea *et al.*, 2012).

In this study, we characterized HS665 and HS666 for specific κ receptor‐mediated *in vivo* behavioural properties following i.c.v. administration in assays of antinociception, locomotor activity and place preference, together with *in vivo* and *in vitro* κ receptor selectivity. The i.c.v. route was selected in order to understand the contribution of activation of central κ receptors in pain suppression, and the prospect of dissociating the beneficial (antinociception) effects of HS665 and HS666 from κ receptor‐mediated adverse effects. Furthermore, their ability to bias toward G protein signalling over β‐arrestin2 interactions with the κ receptor in transfected cellular systems was evaluated.

## Methods

### Animals

All animal studies were preapproved by the Torrey Pines Institute for Molecular Studies (Port St. Lucie, FL, USA), in accordance with the National Institute of Health Guide for the Care and Use of Laboratory Animals. Animal studies are reported in compliance with the ARRIVE guidelines (Kilkenny *et al.*, 2010; McGrath and Lilley, 2015). Sample sizes (i.e. number of animals) were not predetermined by a statistical method, and animals were assigned to groups randomly. Drug treatment experiments were conducted in a blinded fashion. No animals were excluded from statistical analysis.

A total of 202 adult male wild‐type C57BL/6J mice (20–25 g) were obtained from Jackson Laboratory (Bar Harbor, ME, USA). C57BL/6J mice are established and validated subjects in the tail withdrawal, rotorod and place conditioning assays. Twenty‐four male κ receptor gene‐disrupted ‘knockout’ (κ‐KO) mice and 24 male μ receptor gene‐disrupted ‘knockout’ (μ‐KO) mice were obtained from colonies established at the Torrey Pines Institute for Molecular Studies from homozygous breeding pairs of mice backcrossed to C57BL/6J inbred mice for at least 12 generations and obtained from the Jackson Laboratory. Food pellets and distilled water were available *ad libitum.* All mice were tested at 8–12 weeks of age and were group‐housed in ventilated cages (maximum of four animals per cage) in a temperature‐controlled specific pathogen‐free room kept on a 12 h light–dark cycle. Upon the completion of testing, all mice were killed by inhalation of carbon dioxide, followed by cervical dislocation as a secondary measure, as recommended by the American Veterinary Medical Association.

### Antinociceptive testing

The 55°C warm‐water tail withdrawal assay was performed in C57BL/6J wild‐type mice as previously described (McLaughlin *et al.*, 1999), with the latency of the mouse to withdraw its tail from the water taken as the end point. A cut‐off time of 15 s was used in this assay. This model of pain in mice is a well‐established and commonly used model for acute thermal nociception (Le Bars *et al.*, 2001). Following baseline measurements, animals received i.c.v. injection of vehicle (50% DMSO in 0.9% saline for HS665 and HS666 or saline alone for U50,488) or graded doses of test compounds, and the tail withdrawal latency was determined repeatedly every 10 min after drug administration. Separate groups of mice were treated with different doses of the test compound, and individual mice were only used once for antinociceptive testing. To determine the opioid receptor involvement in the antinociceptive activity, κ‐KO and μ‐KO mice were treated with a single dose of test compound and tested as described. Antinociception was calculated according to the following formula: % antinociception = 100 × (test latency − control latency)/(cut‐off − control latency).

### Rotorod assay to determine locomotor activity

Possible sedative or hyperlocomotor effects of test compounds were assessed in C57BL/6J wild‐type mice by rotorod performance, as modified from previous protocols (Eans *et al.*, 2013). The rotorod assay is a well‐established model in mice for assessing a loss of coordinated locomotion and sedation (Kieffer, 1999). Briefly, following seven habituation trials (the last utilized as a baseline measure of rotorod performance), vehicle (50% DMSO in 0.9% saline) or test compound was administered i.c.v. to mice, and mice were assessed after 10 min in accelerated speed trials (180 s max. latency at 0–20 r.p.m.) every 10 min intervals over a 60 min period. Decreased latencies to fall in the rotorod test indicate impaired motor performance.

### Place conditioning to determine CPP/CPA

Conditioned place preference (CPP) is a well‐established model in mice, where drugs with abuse liability such as morphine produce CPP (Bardo *et al.*, 1995) and dysphoric drugs produce aversion (Shippenberg and Herz, 1986). C57BL/6J wild‐type mice were subjected to a counterbalanced place conditioning paradigm, where the compartment in which the animal receives vehicle or drug is randomly assigned regardless of initial preference, using similar timing as detailed previously (Aldrich *et al.*, 2014). The time the animals spent in the middle and two outer compartments were measured as the mice moved freely for 30 min through boxes outfitted with infrared beams (San Diego Instruments, San Diego, CA, USA). Prior to place conditioning, the animals did not demonstrate significant differences in their time spent exploring the outer left (537 ± 14.8 s) versus right (545 ± 14.7 s) compartments. Daily for the next 2 days, vehicle was administered to mice (0.9% saline i.c.v.), which were consistently confined in a randomly assigned outer compartment, half of each group in the right chamber and half in the left chamber. Four hours later, mice were administered the respective test compound (i.c.v.) and confined to the opposite compartment for 30 min. On the fourth day (i.e. the day following the last place conditioning pairing), the CPP or conditioned place aversion (CPA) of each mouse was determined by evaluating time spent in each chamber while the animal again moved freely through the apparatus for 30 min.

### Cell culture

CHO cells stably expressing human κ , μ or NOP receptors (CHO‐hκ , CHO‐hμ and CHO‐hNOP cell lines) were grown in DMEM supplemented with FBS (10%), penicillin/streptomycin (0.1%), l‐glutamine (2 mM) and geneticin (400 μg·mL^−1^). Cell cultures were maintained at 37°C in 5% CO_2_ humidified air.

### [^3^H]‐NOP receptor binding assay

Binding to cell membranes was conducted in a 96‐well format, as described previously (Khroyan *et al.*, 2009). Briefly, CHO‐hNOP cells grown at confluence were removed from the culture plates by scraping, homogenized in 50 mM Tris buffer (pH 7.5) using a Polytron homogenizer, then centrifuged once and washed by an additional centrifugation at 27 000× *g* for 15 min at 4°C. The final pellet was resuspended in Tris buffer, and cell membranes (15 μg) were incubated with [^3^H]‐N/OFQ (0.2 nM) and various concentrations of test compound in a total volume of 1.0 mL for 60 min at 25°C. Non‐specific binding was determined in the presence of 1 μM unlabelled nociceptin/orphanin FQ (N/OFQ). The reactions were terminated by filtration using a Tomtec 96 harvester (Orange, CT, USA) through glass‐fibre filters. Bound radioactivity was counted on a Pharmacia Biotech β‐plate liquid scintillation counter (Piscataway, NJ, USA). All compounds were run in parallel assays in triplicate for comparison.

### [^35^S]‐GTPγS binding assay

The binding of [^35^S]‐GTPγS to membranes from CHO cells stably expressing human κ or μ receptors was conducted as described previously (Khroyan *et al.*, 2009; Spetea *et al.*, 2012). Cells were scraped from culture dishes into 20 mM HEPES with 1 mM EDTA buffer and then centrifuged at 500× *g* for 10 min. Cells were resuspended in the same buffer and homogenized using a Polytron homogenizer. The homogenate was centrifuged at 27 000× *g* for 15 min at 4°C and the pellet resuspended in buffer A containing 20 mM HEPES, 10 mM MgCl_2_ and 100 mM NaCl (pH 7.4). The suspension was recentrifuged at 27 000× *g* and resuspended in buffer A. For the binding assay, membranes (8–15 μg protein) were incubated with [^35^S]‐GTPγS (0.05 nM), GDP (10 nM), and test compounds, in a total volume of 1.0 mL for 60 min at 25°C. Non‐specific binding was determined using 10 μM GTPγS, and the basal binding was determined in the absence of test ligand. Samples were filtered over glass‐fibre filters and counted as described for the receptor binding assays. All compounds were run in parallel assays in triplicate for comparison.

### β‐Arrestin2 recruitment assay

The measurement of κ receptor‐stimulated β‐arrestin2 recruitment was performed using the DiscoveRx PathHunter® eXpress β‐arrestin2 assay (DiscoveRx, Birmingham, UK) according to the manufacturer's protocol and published procedures (Zhou *et al.*, 2013; Brust *et al.*, 2016). In brief, U2OS cells stably co‐expressing the κ receptor and the enzyme acceptor‐tagged β‐arrestin2 fusion protein (U2OS–hκ receptor–β‐arrestin2 cells) were seeded in cell plating medium into 384‐well plates at a density of 2000 cells in 20 μL per well and maintained for 48 h at 37°C. After incubation of the samples with various concentrations of test compound in PBS for 3 h at 37°C, the detection mix was added and incubation was continued for an additional 60 min at room temperature. Chemiluminescence was measured using the Victor2 1420 Multilabel Counter (Perkin Elmer Inc., Waltham, MA, USA). All compounds were run in parallel assays in duplicate for comparison.

### Data and statistical analysis

The data and statistical analysis comply with the recommendations on experimental design and analysis in pharmacology (Curtis *et al.*, 2015). All data are presented as mean ± SEM, with a significance set at *P* < 0.05. All data were statistically evaluated and graphically processed using GraphPad Prism 6.0 software (GraphPad Prism Software Inc., San Diego, CA, USA). To analyse *in vitro* assays, the inhibition constant K_i_ (nM), potency EC_50_ (nM) and efficacy E_max_ (%) values were determined from concentration–response curves by nonlinear regression analysis. In radioligand binding assays, data are normalized to the percentage of specific binding of [^3^H]‐N/OFQ. In the [^35^S]‐GTPγS binding and β‐arrestin2 recruitment assays, efficacy was determined relative to the reference κ or μ receptor full agonists, U69,593 or DAMGO respectively. The Black–Leff operational model (Black and Leff, 1983) was used to calculate the relative affinity transduction coefficients log(τ/K_A_) and bias factors as described previously (van der Westhuizen *et al.*, 2014; Stahl *et al.*, 2015), where the A is the molar concentration of the compound, K_A_ is the equilibrium dissociation constant and τ is the agonist efficacy. The within signalling assay differences were determined by subtracting the log(τ/K_A_) for U69,593 (the reference ligand) from the values obtained for test compounds, which generated the Δlog(τ/K_A_). To determine bias between assays, we calculated relative bias factors by subtracting the Δlog(τ/K_A_) for each assay, as ΔΔlog(τ/K_A_)_path1–path2_. All *in vitro* experiments were performed in multiple replicates, with at least three independent experiments unless otherwise indicated. Determinations of *in vitro* assay performance have previously demonstrated the robustness. No outlier data were removed. No statistical analysis has been performed in datasets of less than five independent experiments and variability of the procedure, which is sufficient for this number of independent experiments. For the evaluation of significant differences in the *in vitro* determinations, a one‐way ANOVA followed by Tukey's multiple comparison *post hoc* test was performed. In the tail withdrawal assays data were normalized to compensate for the baseline response of each individual mouse. Antinociceptive potencies are reported as the effective dose producing 50% antinociception (ED_50_). All dose–response lines were analysed by regression and ED_50_ values and 95% confidence intervals (95% CIs) determined using individual data points. The rotorod data are expressed as the % change from baseline performance, a standard normalization that compensates for each individual animal's baseline response. CPP data are reported as the difference in time spent in the drug‐ and vehicle‐paired compartments. Data from *in vivo* behavioural experiments were evaluated with Student's *t*‐test or one‐way or repeated measures two‐way ANOVA with Tukey's multiple comparison test. Each experimental group included at least eight mice, unless otherwise indicated.

### Materials

The two κ receptor ligands HS665 and HS666 were synthesized and isolated as hydrochloride salts as described previously (Spetea *et al.*, 2012). The radioligand [^35^S]‐GTPγS (1250 Ci·mmol^−1^) was purchased from PerkinElmer (Boston, MA, USA). Unlabelled N/OFQ and [^3^H]‐N/OFQ (120 Ci·mmol^−1^) were obtained from the NIDA drug supply programme. The opioid ligands, DAMGO, U50,488, U69,593 and nor‐binaltorphimine (nor‐BNI), all cell culture media and supplements, and all other chemicals and reagents were purchased from Sigma‐Aldrich (St. Louis, MO, USA). For *in vitro* experiments, test compounds were prepared as 1 mM stocks in 1% DMSO (HS665 and HS666) or water (U69,593, N/OFQ and nor‐BNI) and further diluted to working concentrations in the appropriate medium. For *in vivo* experiments, HS665 and HS666 were dissolved daily prior to use initially in DMSO, and sufficient warm (40°C) sterile saline (0.9%) was then added so that the final vehicle for *in vivo* administration consisted of 50% DMSO in 0.9% saline. U50,488 and morphine sulfate were dissolved in 0.9% sterile saline. The i.c.v. injections, an established route to administer drugs to the CNS, were made directly into the lateral ventricle according to the modified method of Haley and McCormick (1957) as described previously (McLaughlin *et al.*, 1999). Prior to injection, mice were lightly anaesthetized with isoflurane (0.4%), a small (< 0.3 cm) incision was made in the scalp and the injection was made 2 mm lateral and 2 mm caudal to bregma at a depth of 3 mm. Mice typically recover consciousness within 30 s. The volume of all i.c.v. injections was 5 μL; a 10 μL Hamilton microlitre syringe was used for this injection.

### Nomenclature of targets and ligands

Key protein targets and ligands in this article are hyperlinked to corresponding entries in http://www.guidetopharmacology.org, the common portal for data from the IUPHAR/BPS Guide to Pharmacology (Southan *et al.*, 2016), and are permanently archived in the Concise Guide to Pharmacology 2015/16 (Alexander *et al.*, 2015).

## Results

### Antinociceptive effects of HS665 and HS666 after i.c.v. administration in the 55°C warm‐water tail withdrawal assay

**Figure 2:**
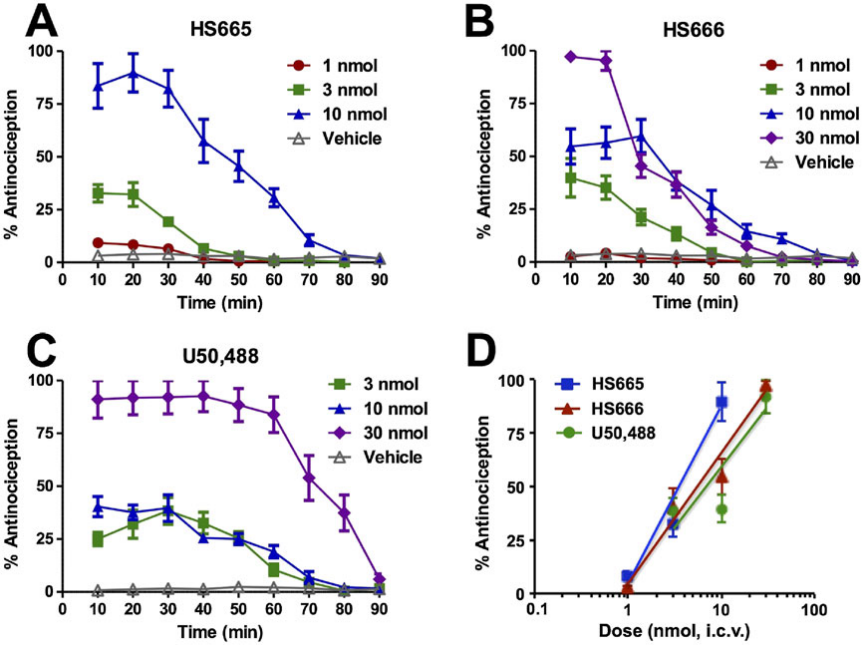
Antinociceptive activity of HS665 and HS666 after i.c.v. administration in the 55°C warm‐water tail withdrawal assay in C57BL/6J mice. Antinociceptive effect of (A) HS665 at doses of 1 (*n* = 8), 3 (*n* = 8) and 10 nmol (*n* = 8); (B) HS666 at doses of 1 (*n* = 8), 3 (*n* = 8), 10 (*n* = 12) and 30 nmol (*n* = 8); and (C) U58,488 at doses of 3 (*n* = 8), 10 (*n* = 8) and 30 nmol (*n* = 8), and vehicle controls (50% DMSO in 0.9% saline for HS665 and HS666, *n* = 8, or saline for U50,488, *n* = 8) with repeated measurements over time. (D) Dose‐dependent antinociceptive effects of HS665, HS666 and U50,488 at peak response. Data are shown as mean % antinociception ± SEM.

The two κ receptor ligands, HS665 and HS666, were initially evaluated for their antinociceptive effects in a model of acute thermal nociception, the 55°C warm‐water tail withdrawal assay, in C57BL/6J mice following i.c.v. administration. Their antinociceptive activity was compared with the reference κ agonist U50,488 (Figure 2). All three agonists increased tail withdrawal latencies to thermal stimulation over the baseline response over time. The onset of the antinociceptive response produced by HS665 and HS666 was rapid (Figure 2A, B). This effect dose‐dependently declined thereafter and returned to values not significantly different from baseline at 90 (1.97 ± 1.18% antinociception for HS665) or 80 min (1.00 ± 0.48% antinociception for HS666) after i.c.v. administration of their highest respective doses. The antinociceptive activity of U50,488 remained largely constant from 10 to 60 min, with detected loss of antinociception 90 min after the largest dose tested (30 nmol i.c.v.; Figure 2C). The i.c.v. administration of HS665 and HS666 generated dose‐dependent increases in mouse tail withdrawal latencies (Figure 2D), resulting in peak antinociceptive ED_50_ (and 95% CI) values of 3.74 (2.98–4.78) at 20 min and 6.02 (4.51–8.08) nmol at 10 min respectively. The i.c.v. doses required to elicit a 50% antinociceptive effect (ED_50_ and 95% CI) for each test compound were calculated at different time points and are presented in Supporting Information Table S1. HS665 was slightly more potent than HS666 in inducing an antinociceptive effect based on the calculated ED_50_ values at the peak effect. On the other hand, when compared with U50,488, which displayed a peak antinociceptive ED_50_ (and 95% CI) value of 7.21 (4.02–11.1) nmol at 30 min, HS665 had increased potency, while HS666 produced similar antinociception.

### The κ receptor but not the μ receptor is involved in the antinociceptive activity of HS665 and HS666 in the 55°C warm‐water tail withdrawal assay

**Figure 3:**
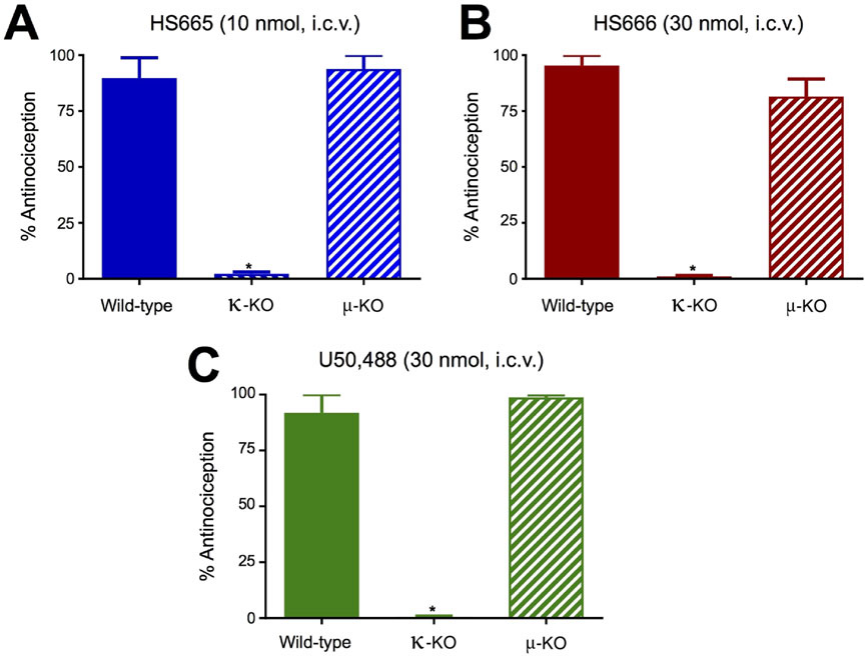
Opioid receptor selectivity of the antinociceptive activity of HS665, HS666 and U50,488 after i.c.v. administration in the 55°C warm‐water tail withdrawal assay in C57BL/6J mice. Antinociceptive activity of (A) HS665 (10 nmol, *n* = 8), (B) HS666 (30 nmol, *n* = 8) and (C) U50,488 (30 nmol, *n* = 8) measured at 20 min was completely absent in κ‐KO mice but not altered in μ‐KO mice when compared with wild‐type mice. Data are shown as mean % antinociception ± SEM. Significantly different from response in the wild‐type mice, *P < 0.05, one‐way ANOVA followed by Tukey's *post hoc* test.

To establish the opioid receptor involved in the observed antinociception induced by HS665 and HS666 following i.c.v. administration, behavioural studies were conducted with κ‐KO and μ‐KO mice. The κ‐KO mice receiving i.c.v. HS665 or HS666 showed no antinociceptive activity when compared with wild‐type mice (Figure 3A, B respectively). In contrast, the antinociceptive activity of HS665 and HS666 in μ‐KO mice was not statistically altered when compared with that in wild‐type animals (Figure 3A, B), indicating that HS665‐ and HS666‐induced antinociception is mediated by κ but not μ receptors. These results were consistent with control testing of the standard κ receptor agonist, U50,488, (30 nmol, i.c.v.) in κ‐KO and μ‐KO mice. While κ‐KO mice showed a significant loss of U50,488 antinociception, the response of μ‐KO mice did not differ from wild‐type C57BL/6J mice (Figure 3C).

### The i.c.v. administration of HS665 and HS666 does not alter locomotor activity

**Figure 4:**
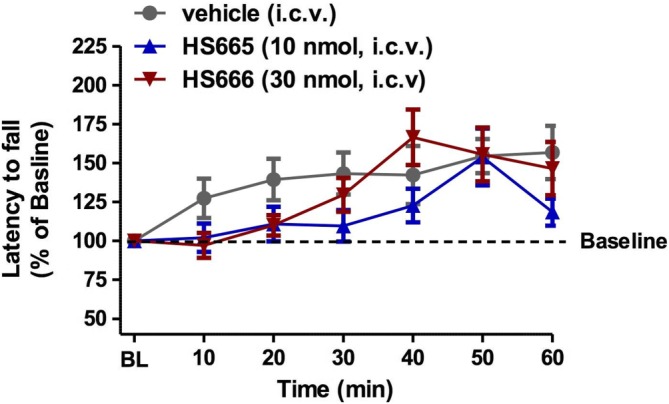
Locomotor activity of HS665 and HS666 after i.c.v. administration to C57BL76J mice in the rotorod assay. Mice received i.c.v. vehicle (50% DMSO on 0.9% saline, *n* = 18), HS665 (10 nmol, *n* = 8) or HS666 (30 nmol, *n* = 8) and were tested on the rotorod apparatus by repeated measurements over time. Latencies to fall are given as the mean % change from baseline (100%) performance ± SEM.

The behavioural effects of κ receptor ligands HS665 and HS666 on evoked locomotor activity were assessed in mice after i.c.v. administration using the rotorod assay. Mice were administered the respective compounds at a dose corresponding to that required to produce the 90% antinociceptive response (ED_90_). Control mice were treated i.c.v. with vehicle (50% DMSO in 0.9% saline). As shown in Figure 4, HS665 and HS666 did not significantly impact motor performance at any time point.

### The i.c.v. administration of HS666 does not produce CPP or CPA

**Figure 5:**
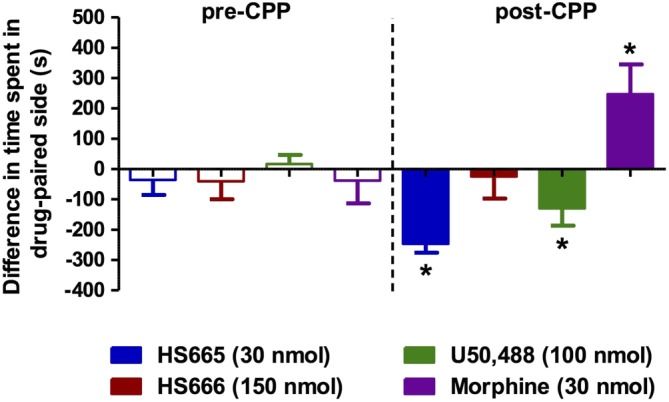
Place conditioning evaluation of HS665 and HS666 in C57BL/6J mice after i.c.v. administration. Following determination of initial preconditioning preferences, mice were place‐conditioned daily for 2 days with HS665 (30 nmol, *n* = 16), HS666 (150 nmol, *n* = 16), U50,488 (100 nmol, *n* = 23) or morphine (30 nmol, *n* = 13). Mean differences in time spent on the drug‐paired side ± SEM are presented. Significantly different from matching preconditioning preference, *P < 0.05, two‐way ANOVA followed by Tukey's *post hoc* test.

The propensity of HS665 and HS666 to induce CPP or CPA was evaluated in mice following i.c.v. administration using the place conditioning paradigm. Mice were administered the test compounds HS665 (30 nmol) or HS666 (150 nmol), corresponding to a dose five times the antinociceptive ED_90_ (ED_90x5_). Additional mice were treated i.c.v. with U50,488 (100 nmol) or morphine (30 nmol) for comparison. As expected, the μ receptor agonist morphine was reinforcing and produced significant CPP, whereas the κ receptor agonists U50,488 and HS665 produced significant place aversion when compared with preconditioning preferences (Figure 5). Of interest, HS666 demonstrated neither significant preference nor aversion in mice after i.c.v. administration of 150 nmol i.c.v. This dose is 25 times higher than the analgesic ED_50_ dose (6.02 nmol i.c.v.) in the 55°C warm‐water tail withdrawal assay.

### HS665 and HS666 show no specific binding to the human NOP receptor

We have reported previously specific binding of HS665 and HS666 to the three classical opioid receptors, κ , μ and δ, with both ligands having high affinity and selectivity for the human κ receptor (Spetea *et al.*, 2012). Binding affinities of HS665 and HS666 were 0.49 and 5.90 nM, respectively, for the κ receptor, 542 and 826 nM, respectively, for the μ receptor, and >10 000 nM for both for the δ receptor. Competitive inhibition of [^3^H]‐N/OFQ binding to the NOP receptor by HS665 and HS666 was performed using *in vitro* radioligand binding assays with membranes from CHO cells expressing the human NOP receptor. HS665 and HS666 displayed no significant affinity for the NOP receptor up to a concentration of 10 μM. In the same assay, the endogenous N/OFQ ligand had high binding affinity (K_i_ = 0.16 ± 0.04 nM) for the NOP receptor.

### HS665 and HS666 do not induce μ receptor‐mediated G protein activation and are specific in promoting G protein signalling through the κ receptor

**Figure 6:**
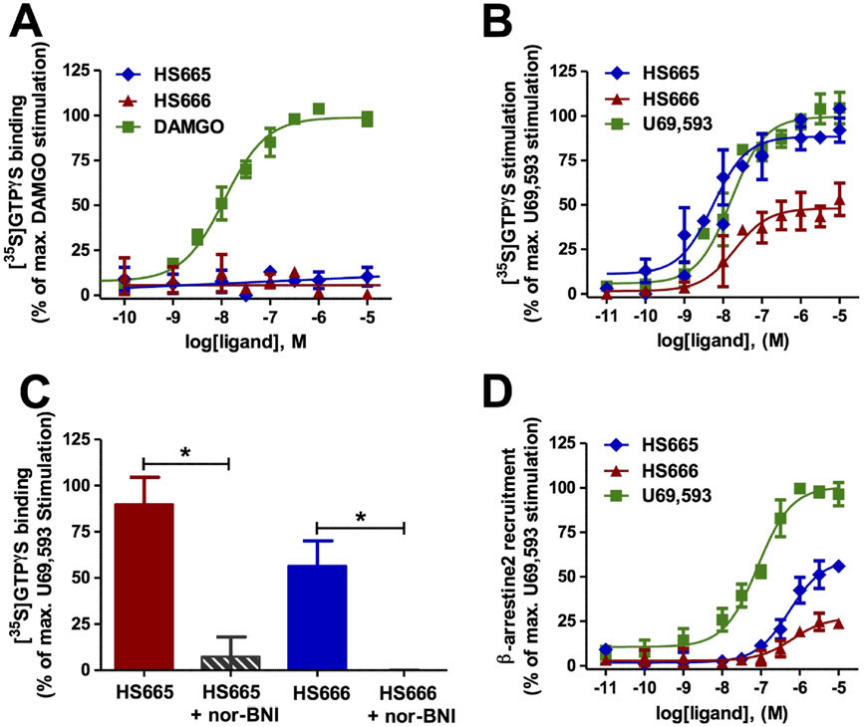
*In vitro* functional activity of HS665 and HS666. (A) Stimulation of [^35^S]‐GTPγS binding to the human μ receptor by HS665, HS666 and DAMGO determined in the [^35^S]‐GTPγS binding assay with membranes from CHO‐hμ receptor cells. The data were normalized to the maximum stimulation caused by DAMGO (100%) (*n* = 3 independent experiments). (B) Stimulation of [^35^S]‐GTPγS binding to the human κ receptor by HS665, HS666 and U69,593 determined in the [^35^S]‐GTPγS binding assay with membranes from CHO‐hκ receptor cells. The data were normalized to the maximum stimulation caused by U69,593 (100%) (*n* = 5 independent experiments). (C) Selective κ receptor‐mediated G protein activation by HS665 and HS666. Significant antagonism by nor‐BNI (0.1 μM) of the [^35^S]‐GTPγS binding stimulated by HS665 (1 μM) or HS666 (1 μM) determined in the [^35^S]‐GTPγS binding assay with membranes from CHO‐hκ receptor cells. The data were normalized to the % stimulation induced by 10 μM U69,593 (100%) (*n* = 5 independent experiments). Significantly different from the stimulation with HS665 or HS666 alone, *P < 0.05, one‐way ANOVA followed by Tukey's *post hoc* test. (D) Concentration–response curves of HS665, HS666 and U69,593 for β‐arrestin2 recruitment to the human κ receptor expressed in U2OS–β‐arrestin2 cells using the PathHunter β‐arrestin2 assay. Responses were normalized to the maximum effect of U69,593 (100%) (*n* = 3 independent experiments). All data are presented as the mean ± SEM.

Ligand regulation of the binding of [^35^S]‐GTPγS is one of the most widely used methods to measure receptor activation of heterometric G protein (Bohn *et al.*, 2015) and has been commonly used as a functional measure *in vitro* for determination of potencies and efficacies of compounds when a reference ligand is defined as a full agonist (Traynor and Nahorski, 1995; Zhu *et al.*, 1997). *In vitro* studies were carried out to evaluate the functional activity of HS665 and HS666 at the μ receptor using the [^35^S]‐GTPγS binding assay with membranes from CHO cells expressing the human μ receptor. Neither of the two κ ligands induced G protein activation at the μ receptor, at concentrations up to 10 μM, consequently showing no agonist activity at this receptor, in contrast to the high potency (EC_50_ of 14.1 ± 5.8 nM) and stimulating effect shown by the reference full μ receptor agonist DAMGO (Figure 6A). This result was consistent with the low affinity at the μ receptor reported earlier (K_i_ values of 542 nM for HS665 and 826 nM for HS666; Spetea *et al.*, 2012) and the present observation on their persistent antinociceptive activity in μ‐KO mice (Figure 3A, B). As shown in Figure 6B, HS665, HS666 and U69,593 produced a concentration‐dependent increase in [^35^S]‐GTPγS binding to membranes from CHO cells expressing the human κ receptor, with HS665 having maximal stimulation similar to the full agonist U69,593 and HS666 showing partial agonist activity. Agonist potencies (ED_50_) and maximal response (% E_max_) values are listed in Table 1. The classification of HS666 as a partial agonist was further confirmed by its partial inhibition of U69,593‐stimulated G protein coupling as compared with full antagonism produced by nor‐BNI (Supporting Information Figure S1). Their functional activity at the δ receptor was not evaluated due to the earlier findings regarding the absence of specific binding to this receptor using radioligand binding assays (Spetea *et al.*, 2012).

**Table 1:** Potencies and efficacies at the human κ receptor of HS665 and HS666 in comparison with U69,593 in functional *in vitro* assays for G protein activation and β‐arrestin2 recruitment

Compound	G protein coupling^a^		β‐arrestin2 recruitment^b^	
	EC_50_ (nM)	E_max_ (%)^c^	EC_50_ (nM)	E_max_ (%)^c^
HS665	4.98 ± 1.29	88 ± 3	463 ± 101	55 ± 1
HS666	35.7 ± 6.0	50 ± 4	449 ± 56	24 ± 1
U69,593	18.2 ± 2.9	100	67.7 ± 10.7	100

a Measured in the [^35^S]‐GTPγS binding assay with membranes from CHO cells stably expressing the human κ receptor (*n* = 5 independent experiments).

b Measured in the PathHunter β‐arrestin2 recruitment assay with U2OS cells co‐expressing the human κ receptor and the enzyme acceptor tagged β‐arrestin2 fusion protein (*n* = 3 independent experiments).

c E_max_ (%) are percentage relative to the maximal effect of U69,593 (100%). All values are presented as the mean ± SEM.

Additional *in vitro* functional activity investigations established that the κ receptor is specifically involved in the G protein coupling induced by HS665 and HS666. For this, we assessed the ability of the selective κ antagonist, nor‐BNI, to reverse the agonist‐induced [^35^S]‐GTPγS binding by incubating HS665 (1 μM) or HS666 (1 μM) in the absence or in the presence of nor‐BNI (0.1 μM). As shown in Figure 6C, the stimulating effects of both ligands on G protein activation were significantly antagonized by nor‐BNI, indicating a κ receptor‐mediated G protein signalling.

### HS665 and HS666 biased κ receptor signalling toward G protein coupling over β‐arrestin2 recruitment

In addition to G protein coupling, another important signalling event following κ receptor stimulation is agonist‐induced β‐arrestin2 recruitment. Increasing evidence suggests that this signalling may significantly mediate the negative side effects associated with κ receptor activation (Bruchas *et al.*, 2007). To investigate their ability to promote κ receptor‐mediated β‐arrestin2 signalling, HS665 and H666 were profiled for their potency and efficacy in the PathHunter β‐arrestin2 recruitment assay with U2OS cells co‐expressing the human κ receptor and the enzyme acceptor tagged β‐arrestin2 fusion protein. In this assay, test compounds were examined in parallel with U69,593, which served as the reference full κ receptor agonist.

Each κ ligand produced concentration‐dependent activation of β‐arrestin2, although with distinct functional profiles (Figure 6D). In contrast to U69,593, which robustly recruited β‐arrestin2 to the κ receptor, HS665 and HS666 were significantly (approximately sevenfold) less potent in inducing interactions between β‐arrestin2 and the κ receptor (Table 1). Additionally, the two κ ligands displayed much lower efficacy for β‐arrestin2 recruitment than U59,593, with 55% for HS665 and only 24% for HS666 of the efficacy of U69,593.

**Figure 7:**
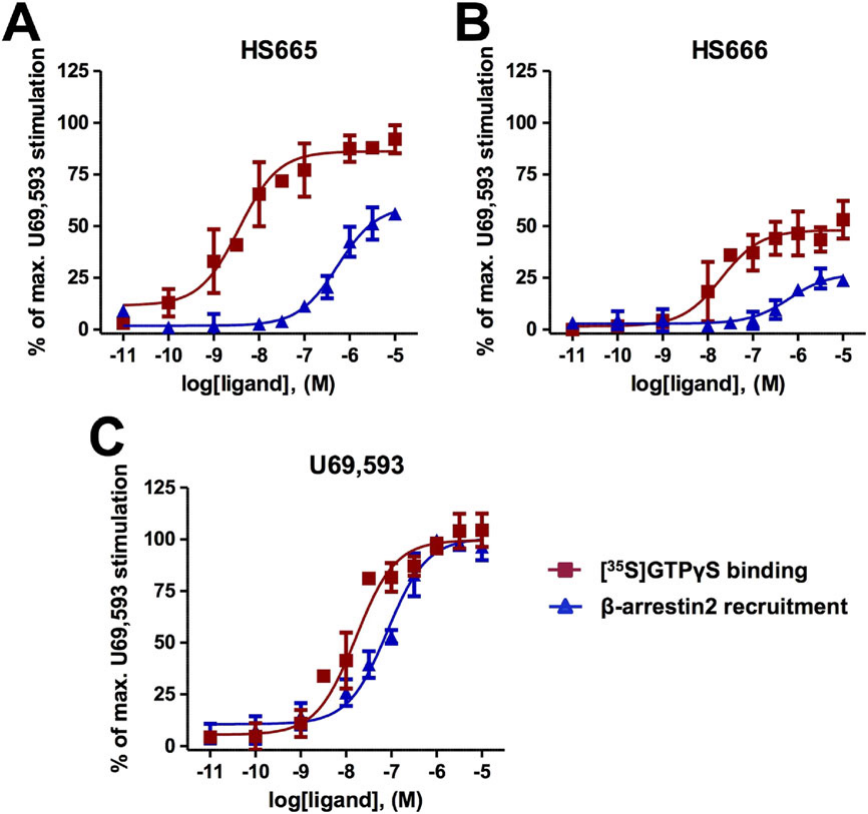
Concentration‐dependent curves of agonist‐stimulated G protein coupling (the [^35^S]‐GTPγS binding assay, *n* = 5 independent experiments) and β‐arrestin2 recruitment (the DiscoveRx PathHunter β‐arrestin2 assay, *n* = 3 independent experiments) to the human κ receptor by (A) HS665 and (B) HS666, in comparison with (C) U69,593. Responses were normalized to the maximum effect of U69,593 (100%). All values are presented as the mean ± SEM.

To examine whether HS665 and HS666 display bias toward the activation of G protein‐ over β‐arrestin2‐mediated signalling, we compared their functional activity, that is, potency and efficacy, across two functional assays that measure G protein coupling (using the [^35^S]‐GTPγS binding assay) and β‐arrestin2 recruitment (using the DiscoveRx PathHunter β‐arrestin2 assay) at the human κ receptor (Figure 7 and Table 1). When compared with the standard κ agonist U69,593 (Figure 7C), HS665 elicits weak partial agonism for β‐arrestin2 recruitment, while being highly potent and fully efficacious in promoting κ receptor‐dependent responses indicative of G protein activation. As shown in Figure 7A, there was >90‐fold rightward shift in the concentration–response curve of HS665 in the [^35^S]‐GTPγS assay versus PathHunter assay, paralleled by an almost twofold reduction in agonist efficacy. Although it acts as an agonist with high G protein coupling potency, HS666 fails to robustly engage β‐arrestin2 recruitment, depicted by the marked 13‐fold decrease in potency and extremely low efficacy in promoting β‐arrestin2‐κ receptor interaction (Figure 7B, Table 1). Using the two signalling assays, we calculated the bias factors using the operational model (van der Westhuizen *et al.*, 2014; Stahl *et al.*, 2015). Compared with U69,593, which was normalized to a value of 1, the bias factors for HS665 and HS666 were 389 and 62 respectively. The transduction ratio (log(τ/K_A_)) values for compounds and assays are listed in Supporting Information Table S2. Overall, the present data indicate that the two newly investigated κ ligands activate the κ receptor in a manner that is preferentially biased towards G protein signalling.

## Discussion and conclusions

A growing body of evidence indicates that prototypical κ agonists are effective analgesics with reduced potential for abuse (Lemos and Chavkin, 2011; Cahill *et al.*, 2014). Despite the strength of preclinical data, testing the premise in humans and developing compounds into drugs have been impeded by dysphoria, psychotomimesis, anhedonia and hallucinations. Therefore, new chemical approaches including the design of G protein‐biased κ ligands for creating analgesics with fewer side effects are sought, and such drugs with improved benefit/risk profile are likely to have a significant impact (Dogra and Yadav, 2015; Rankovic *et al.*, 2016).

In this study, we present a thorough exploration of the *in vitro* and *in vivo* κ receptor pharmacology and signalling of two new κ ligands from the class of diphenethylamines, HS665 and HS666. The major finding is that HS666 emerged as a G protein‐biased κ receptor partial agonist displaying analgesia without the typical side effects associated with κ receptor activation by unbiased agonists. We also showed that the full κ agonist HS665 produces antinociception and is aversive in mice after central (i.c.v.) administration, despite its decreased ability to promote κ receptor/β‐arrestin2 interaction.

Our findings from behavioural studies using a mouse model of acute thermal nociception establish that activation of central κ receptors by HS665 and HS666 resulted in a time‐ and dose‐dependent antinociceptive response. HS665 was slightly more potent (lesser than twofold) than HS666 in inducing antinociception (ED_50_ values of 3.74 vs*.* 6.02 nmol i.c.v., respectively). When compared with U50,488, HS665 displayed a two‐fold greater antinociceptive potency, while HS666 showed a similar effect. Moreover, their analgesic potencies were largely comparable with that reported for morphine (ED_50_ of 2.35 nmol, i.c.v.; Aldrich *et al.*, 2014). Using genetic approaches, we demonstrated the *in vivo* κ receptor selectivity of the antinociceptive activity, as the effect of both ligands was absent in κ‐KO mice but still detected in the μ‐KO mice. The κ receptor‐mediated analgesia of i.c.v. HS665 in the tail withdrawal assay is in‐line with our previous *in vivo* pharmacological observations in a mouse model of visceral pain, specifically the blockade by the selective κ antagonist nor‐BNI of the anti‐writhing effect of HS665 after s.c. administration (Spetea *et al.*, 2012).

Exploring possible *in vivo* activation of central κ receptors by HS665 and HS666 on behavioural effects mediated by the κ receptor, we demonstrated that HS665 and HS666 had no effect on motor coordination at doses corresponding to a high analgesic effect in the rotorod assay. This assay has long been used to assess potential liabilities of CNS‐active drugs, including opiates, where the drug effect could be due to a number of factors such as sedation and disrupted motor coordination (Kieffer, 1999). Neuroanatomical, neurochemical and pharmacological data support the key physiological role of the κ receptor expressed in the limbic system in mood and reward. Activation of central κ receptors causes dysphoria in humans (Barber and Gottschlich, 1997) and an aversive response in animals (Land *et al.*, 2008), evidenced by its ability to produce CPA in animals. One of the underlying mechanisms thought to account for the dysphoric effects of κ agonists is their ability to suppress mesolimbic dopamine release within reward circuitry, although other mechanisms were also suggested (Land *et al.*, 2009). In this study, we have evaluated the specific κ receptor‐mediated aversive effects of HS665 and HS666 after i.c.v. administration in mice using the place conditioning behavioural paradigm. Unlike the μ agonist morphine, or the κ agonist U50,488, HS666 produced neither preference nor aversion for the drug‐paired compartment in our place conditioning model at doses 25 times higher than the antinociceptive ED_50_ value. In contrast, HS665 induced aversion when tested at a dose eight times the antinociceptive ED_50_ value.

An important objective of our study was to understand the signalling mechanism behind the *in vivo* activity profiles in mice of the two targeted κ ligands by exploring the role and contribution of G protein signalling pathway in mediating various behavioural responses to activation of central κ receptors. Characterization of molecular pharmacological properties *in vitro* revealed that the diphenethylamines, HS665 and HS666, possessed significant binding affinity at the human κ receptor (Spetea *et al.*, 2012; Guerrieri *et al.*, 2015). These previous outcomes are now extended by the absence of NOP receptor affinity demonstrated herein. Furthermore, our current data are supported by the recent computational findings on molecular docking of HS665 and HS666 to an active‐like structure of the human κ receptor attained by molecular dynamics (Guerrieri *et al.*, 2016). While HS665 demonstrated potent and full κ agonism in an *in vitro* assay of G protein activation, HS666, was a selective κ receptor partial agonist. No indication of their efficacy to promote G protein coupling at the human μ receptor was detected, though this result was not surprising considering the very low affinity at the μ receptor reported earlier (Spetea *et al.*, 2012) and the present observations on their maintained antinociceptive activity in μ‐KO mice.

In addition to canonical G protein‐mediated signalling, the κ receptor can activate G protein‐independent pathways. Principal among these is the β‐arrestin pathway (Bruchas and Chavkin, 2010). Investigations into which signalling pathways contribute to therapeutic efficacy and which contribute to side effects of κ ligands have gained considerable attention over the past few years (Dogra and Yadav, 2015; Rankovic *et al.*, 2016). The evolving concept of biased agonism could provide a new potential for κ receptor‐targeted therapies with fewer unwanted effects, if appropriate ligands can be identified. Until now, only a few examples of G protein‐biased κ ligands were reported from *in vitro* and *in vivo* studies (Rives *et al.*, 2012; Zhou *et al.*, 2013; Maillet *et al.*, 2015; White *et al.*, 2015; Brust *et al.*, 2016; Zangrandi *et al.*, 2016). To determine functional activity to a non‐G protein κ receptor stimulated pathway, the ability of HS665, HS666 and U69,593 to induce β‐arrestin2 binding to the κ receptor was assessed using the PathHunter β‐arrestin2 assay. The experimental data indicate that under our testing conditions, HS666 is a very weak partial agonist of κ receptor signalling through the β‐arrestin2 (24% efficacy). HS666 exhibited significant and potent partial agonism of the κ receptor for G protein coupling, but there is a strong qualitative and quantitative bias in favour of G protein signalling over β‐arrestin2 recruitment in cell lines. Despite its full agonism of G protein activation, HS665 acted as a partial agonist with a 55% efficacy at inducing β‐arrestin2 recruitment to the κ receptor. As expected, the unbiased agonist U69,593 robustly stimulated interactions between β‐arrestin2 and the κ receptor with a full response and potency (EC_50_ of 67.7 nM) comparable to that of previous reports (Schmid *et al.*, 2013; Zhou *et al.*, 2013; Maillet *et al.*, 2015; Brust *et al.*, 2016).

Future evaluation of the contribution of biased signalling toward the differences in behavioural effects of the new κ ligands, HS665 and HS666, in comparison with U50,488 will be essential. Such studies are particularly timely, given the reports that biased signalling at the κ receptor can distinguish the aversive effects of κ agonists from the antinociceptive effects. Elegant previous studies have provided evidence that aversive actions are dependent on the β‐arrestin2 binding to the κ receptor, while G protein signalling regulates analgesia (Bruchas *et al.*, 2007). From this theoretical framework, the G protein‐biased κ receptor agonism shown by HS666 in *in vitro* signalling assays is predictive of its potent and efficacious antinociception without aversion and motor impairment, consistent with its weak ability to activate the β‐arrestin2 pathway. In contrast, U50,488 and other unbiased full κ receptor agonists such as U69,593 and salvinorin A produce pronounced aversive effects. Moreover, as all test compounds showed similar antinociceptive activity and were administered centrally (i.c.v.), the differences between U50,488 and HS666 in the CPA model do not simply reflect dissimilarities in CNS penetration between the drugs but rather further implicate the biased signalling profile of HS666.

HS665 elicits marked antinociception with no motor incoordination while inducing aversive‐like actions in the CPA assay in mice. This is a notable profile, as HS665 appears to activate favourably G protein coupling to the human κ receptor over β‐arrestin2 recruitment, though still with higher efficacy at β‐arrestin2 recruitment and a bias factor than HS666. Consistent with this, a recent report showed that the structural analogue of salvinorin A, RB‐64, was characterized *in vitro* as a G protein‐biased κ receptor agonist (with full efficacy for G protein and β‐arrestin2 activation, but low potency for β‐arrestin2. RB‐64 is aversive, yet lacking sedative or coordination‐impairing actions in mice. On further investigation, some aversive activity of RB‐64 was still detected in β‐arrestin2 KO mice (White *et al.*, 2015). This may indicate that aversion in some compounds is related to the efficacy in G protein coupling with full agonist activity leading to aversion‐like activity but partial agonist activity, as in the case of HS666, inducing no dysphoria. Alternatively, as previous reports link the activation of p38 MAPK to κ receptor‐induced aversion presumed to be mediated by β‐arrestin2 signalling (Bruchas *et al.*, 2007), our results with HS665 may suggest that other factors such as p38 may contribute to κ receptor‐dependent aversion. Activation of p38 MAPK was not addressed in our study, and we did not answer the question whether p38 MAPK could be activated by an alternative signalling mode or if the aversion may be induced by a p38 MAPK‐independent manner. Furthermore, differences between human and mouse κ receptors might exist in terms of functional selectivity (i.e. β‐arrestin recruitment) (Schattauer *et al.*, 2012), and the translatability of data is always an issue across *in vitro* cellular systems to animal models (Zhou and Bohn, 2014).

In conclusion, our data strongly indicate that the two κ ligands, HS665 and HS666, activate central κ receptors to produce analgesia and other behavioural effects. HS666 appears to carry the prerequisite pharmacological characteristics as an analgesic κ drug with reduced liability for aversive effects, potentially due to its low efficacy in the β‐arrestin2 signalling pathways *in vitro*. These results offer valuable structural and functional insights into the design and/or discovery of drugs with improved pharmacological profiles and enhanced therapeutic efficacies for the treatment of pain and other human disorders.

## Author contributions

M.S. designed the research study, performed the research, analysed the data and contributed to the writing of the manuscript. S.O.E. performed the research. M.L.G. performed the research. A.L. performed the research and analysed the data. M.M. performed the research and analysed the data. L.T. designed the research study, contributed essential reagents or tools and contributed to the writing of the manuscript. H.S. designed the research study, contributed essential reagents or tools and contributed to the writing of the manuscript. J.P.M. designed the research study, analysed the data and contributed to the writing of the manuscript.

## Conflict of interest

The authors declare no conflicts of interest.

## Declaration of transparency and scientific rigour

This Declaration acknowledges that this paper adheres to the principles for transparent reporting and scientific rigour of preclinical research recommended by funding agencies, publishers and other organisations engaged with supporting research.

## Supporting information


**Figure S1** HS666 is a partial agonist for G protein activation in CHO‐hKOR cells. [^35^S]‐GTPγ;S binding was determined using CHO‐hKOR cell membranes following ligand treatment. Membranes were incubated with increasing concentrations of HS666 (*n* = 4) or nor‐BNI (*n* = 4) in the presence or in the absence of U69,593 (1 μM). The data were normalized to the maximum stimulation caused by U69,593 (100%). HS666 partially blocks U69,593‐stimulated [^35^S]‐GTPγS coupling, whereas nor‐BNI completely blocks coupling. Values are reported as the mean ± SEM.
**Table S1** Antinociceptive potency of HS665, HS666 and U50,488 after i.c.v. administration in the 55 °C warm‐water tail‐withdrawal assay.
**Table S2** Analysis of bias comparing G protein signalling and β‐arrestin2 requirement of HS665 and HS666 in comparison to U69,593 activity.
