# Supplementary material for: Selective κ receptor partial agonist HS666 produces potent antinociception without inducing aversion after i.c.v. administration in mice
Source: Br J Pharmacol. 2017 Jun 27;174(15):2444–56. doi: 10.1111/bph.13854 (PMC5513865; doi:10.1111/bph.13854)
Supplement: Supplementary file 1 — Figure S1 HS666 is a partial agonist for G protein activation in CHO‐hKOR cells. [35S]‐GTPγ;S binding was determined using CHO‐hKOR cell membranes following ligand treatment. Membranes were incubated with increasing concentrations of HS666 (n = 4) or nor‐BNI (n = 4) in the presence or in the absence of U69,593 (1 μM). The data were normalized to the maximum stimulation caused by U69,593 (100%). HS666 partially blocks U69,593‐stimulated [35S]‐GTPγS coupling, whereas nor‐BNI completely blocks coupling. Values are reported as the mean ± SEM. Table S1 Antinociceptive potency of HS665, HS666 and U50,488 after i.c.v. administration in the 55 °C warm‐water tail‐withdrawal assay. Table S2 Analysis of bias comparing G protein signalling and β‐arrestin2 requirement of HS665 and HS666 in comparison to U69,593 activity. [file BPH-174-2444-s001.pdf]

Supporting Information

Selective κ opioid receptor partial agonist HS666 produces potent antinociception without inducing aversion after i.c.v. administration in mice

Mariana Spetea[1,2], Shainnel O. Eans[2,3], Michelle L. Ganno[2], Aquilino Lantero[1], Michael Mairegger[1], Lawrence Toll[2], Helmut Schmidhammer[1] and Jay P. McLaughlin[2,3]

[1]Department of Pharmaceutical Chemistry, Institute of Pharmacy and Center for Molecular Biosciences Innsbruck (CMBI), University of Innsbruck, Innsbruck, Austria

[2]Torrey Pines Institute for Molecular Studies, Port St. Lucie, FL, USA

[3]Department of Pharmacodynamics, University of Florida, Gainesville, FL, USA

Corresponding authors

Mariana Spetea

Department of Pharmaceutical Chemistry, Institute of Pharmacy and Center for Molecular Biosciences (CMBI), University of Innsbruck, Innrain 80-82, 6020 Innsbruck, Austria; Tel.: +43 512 507 58277; Fax: +43 512 507 58299; e-mail: Mariana.Spetea@uibk.ac.at

Jay P. McLaughlin

Department of Pharmacodynamics, University of Florida, Gainesville, FL 32610-0487, USA; Tel.: +1 352 273 7207; Fax: +1 352 273 7705; e-mail: JMcLaughlin@cop.ufl.edu

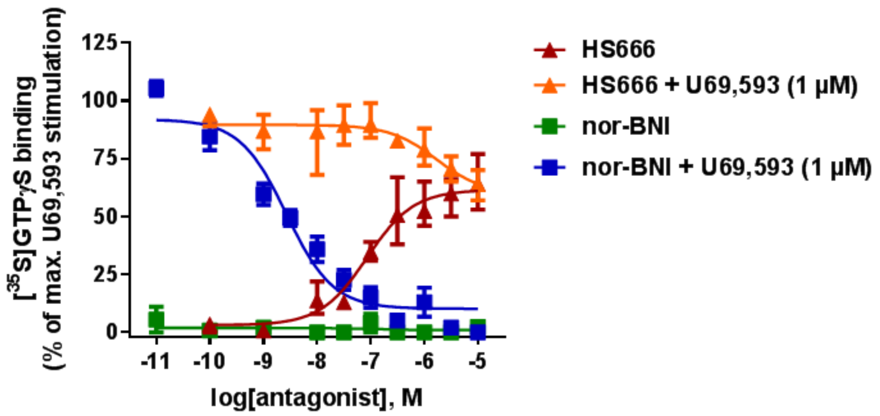

Figure S1. HS666 is a partial agonist for G protein activation in CHO-hKOR cells. [^{35}S]GTPγS binding was determined using CHO-hKOR cell membranes following ligand treatment. Membranes were incubated with increasing concentrations of HS666 (*n* = 4) or nor-BNI (n = 4) in the presence or in the absence of U69,593 (1 µM). The data were normalized to the maximum stimulation caused by U69,593 (100%). HS666 partially blocks U69,593-stimulated [^{35}S]GTPγS coupling, whereas nor-BNI completely blocks coupling. Values are reported as the mean ± SEM.

Table S1. Antinociceptive potency of HS665, HS666 and U50,488 after i.c.v. administration in the 55°C warm-water tail-withdrawal assay

Time (min)	ED_{50} and 95% C.I. (nmol, i.c.v.)[a]		
	**HS665**	**HS666**	**U50,488**
10	3.99 (3.14 – 5.33)	6.02 (4.51 – 8.08)	8.94 (6.55 – 12.0)
20	3.74 (2.98 – 4.78)	6.14 (4.82 – 7.86)	8.31(5.47 – 12.0)
30	4.74 (3.72 – 6.41)	not calculable	7.21 (4.02 – 11.1)
40			9.56 (6.17 – 14.8)
50			11.1 (7.83 –16.3)
60			14.2 (10.7 – 20.4)

[a]Groups of C57Bl/6J mice ($n \geq 8$ per group) were administered the respective compound, and evaluated in the 55°C warm-water tail-withdrawal assay. ED_{50} and 95% confidence interval (C.I.) values were calculated using linear regression and are reported at different time points.

Table S2. Analysis of bias comparing G protein signalling and β-arrestin2 requirement of HS665 and HS666 in comparison to U69,593 activity

Compound	$\log(\tau/K_A)$		$\Delta\log(\tau/K_A)$		$\Delta\Delta\log(\tau/K_A)$	Bias factor
	G protein[a]	β-arrestin2[b]	G protein[a]	β-arrestin2[b]		
HS665	8.36 ± 0.16	4.96 ± 0.12	-2.21	0.38	2.59	389
HS666	5.73 ± 0.29	3.52 ± 0.02	-3.65	-1.86	1.79	62
U69,593	7.98 ± 0.11[c]<br>7.59 ± 0.17[d]	7.17 ± 0.16	0	0	0	1

[a]Derived from the [^{35}S]GTPγS binding assay with membranes from CHO cells stably expressing the human KOP receptor (*n* = 5 independent experiments). [b]Derived from the PathHunter β-arrestin2 recruitment assay with U2OS cells co-expressing the human KOP receptor and the enzyme acceptor tagged β-arrestin2 fusion protein (*n* = 3 independent experiments). [c]Value used for U69,53, when assessed in parallel with HS665 within each experiment. [d]Value used for U69,53, when assessed in parallel with HS666 within each experiment. Transduction coefficients (($\log(\tau/K_A)$, mean ± SEM), and bias factors ($\Delta\Delta\log(\tau/K_A)_{\text{G protein - β-arrestin2}}$) were calculated using the operational model.
